# Dandruff Is Associated with Disequilibrium in the Proportion of the Major Bacterial and Fungal Populations Colonizing the Scalp

**DOI:** 10.1371/journal.pone.0058203

**Published:** 2013-03-06

**Authors:** Cécile Clavaud, Roland Jourdain, Avner Bar-Hen, Magali Tichit, Christiane Bouchier, Florence Pouradier, Charles El Rawadi, Jacques Guillot, Florence Ménard-Szczebara, Lionel Breton, Jean-Paul Latgé, Isabelle Mouyna

**Affiliations:** 1 Unité des Aspergillus, Institut Pasteur, Paris, France; 2 Advanced Research, L’Oréal Research and Innovation, Aulnay sous Bois, France; 3 MAP5, UFR de Mathématiques et Informatique, Université Paris Descartes, Paris, France; 4 PF1, Plateforme génomique, Institut Pasteur, Paris, France; 5 International General Direction of Hair Metiers, L’Oréal Research and Innovation, Saint-Ouen, France; 6 UMR BIPAR, Ecopham, Ecole Nationale Vétérinaire d’Alfort (ENVA),Maisons-Alfort, France; 7 Corporate Microbiology, L’Oréal Research and Innovation, Chevilly Larue, France; California Department of Public Health, United States of America

## Abstract

The bacterial and fungal communities associated with dandruff were investigated using culture-independent methodologies in the French subjects. The major bacterial and fungal species inhabiting the scalp subject’s were identified by cloning and sequencing of the conserved ribosomal unit regions (16S for bacterial and 28S-ITS for fungal) and were further quantified by quantitative PCR. The two main bacterial species found on the scalp surface were *Propionibacterium acnes* and *Staphylococcus epidermidis,* while *Malassezia restricta* was the main fungal inhabitant. Dandruff was correlated with a higher incidence of *M. restricta* and *S. epidermidis* and a lower incidence of *P. acnes* compared to the control population (p<0.05). These results suggested for the first time using molecular methods, that dandruff is linked to the balance between bacteria and fungi of the host scalp surface.

## Introduction

Dandruff is a scalp disorder occurring in about 17–50% of human individuals depending on the population tested [1]–[3]. Dandruff is characterized by an abnormal flaking of the scalp, related to mild inflammatory reaction, where stratum corneum is altered exhibiting disrupted cohesion between the corneocytes and cell hyperproliferation [4]–[6]. Although previous reports have suggested that dandruff was due to the presence of *M. restricta* and *M. globosa* [2], [7]–[9], discrepancies exist between studies: for example, Gemmer *et al.* [9] reported that the most prevalent fungal species found in the dandruff scalps were *M. restricta* and *M. globosa*; according to Paulino *et al*. [10], *M. restricta* was the most abundant *Malassezia* species on the scalp of healthy (non-dandruff) population; recently, Park *et al.* [11] reported that *Filobasidium floriforme* was the main fungal species isolated from dandruff scalps, whereas *Acremonium* spp. was identified in the non-dandruff scalps. In addition to fungi, human scalp is also colonized by bacterial populations [12] but their association with dandruff has not been shown earlier.

Here, in a sample of French population, we identified the major bacterial and fungal species present in the scalps of healthy and dandruff subjects and showed that dandruff is significantly associated with a higher amount of both *M. restricta* and *S. epidermidis* and a lower amount of *P. acnes* in dandruff population compared to control population.

## Results

### Major Species Found on Scalps with or without Dandruff

**Figure 1:**
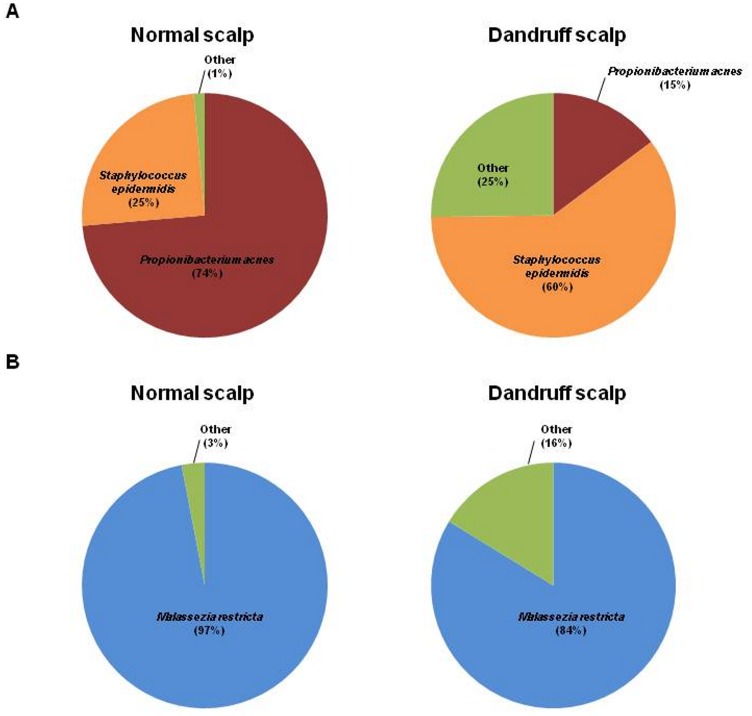
Distribution of bacterial and fungal species in 10-normal and 9-dandruff scalps from set-1. (A) Distribution of 2,122 sequences (∼1500 bp) of 16S rDNA and (B) 2,225 sequences (∼1500 bp) of ITS-28S rDNA between the normal scalps and dandruff–affected scalps. Results are presented as the percent (%) of total sequences recovered per species. The predominant species are presented on this figure, while the detailed list of the sequences identified is presented in the Supporting Information (Table S1 and S2).

Culture-independent methods to identify microbial scalp inhabitants have been scarcely used. However, direct characterization of DNA from scalp samples avoids any bias due to the culture step which has been shown to often under-evaluate the count of skin microbial inhabitants especially for *Malassezia* spp. [13], [14]. Cloning and sequencing of the conserved ribosomal unit regions (16S for bacterial and 28S-ITS for fungal) lead to the identification of the major bacterial and fungal scalp colonizers. The two major bacterial genera found in the scalps whatever their dandruff status were: *Propionibacterium* and *Staphylococcus*. These bacterial genera, i.e. *Propionibacteria* and *Staphylococci*, accounted respectively for 49% and 40% of all sequences retrieved. Interestingly, the sequences of *Propionibacterium* spp. consisted in *P. acnes* (99.7%) and *P. granulosum* (0.3%) and the sequences of *Staphylococcus* spp. mainly included *S. epidermidis* (99.1%) and *S. caprae* (0.5%). The remaining 11% of the sequences identified included species present in minor amount (each species representing less than 2% of the total number of bacterial sequences), belonging to the genera *Streptococcus*, *Acinetobacter*, *Corynebacterium*, *Pseudomonas* and *Moraxella* (Figure 1A, Table S1).

*Malassezia restricta* was the major fungal species present on the scalps with or without dandruff (97% and 84% of the sequences obtained from the normal and dandruff subjects, respectively) (Figure 1B, Table S2). *M. globosa* and *M*. *sympodialis* accounted for less than 1% of all sequences retrieved. *M. slooffiae* (4% all the fungal sequences) was found on the scalp of one volunteer only. The remaining sequences identified belonged to the genera *Exophiala*, *Penicillium*, *Rhodotolura* and other *Malassezia* spp. (Table S2).

### The Presence of Dandruff is Associated with Disequilibrium in the Proportion of the Major Bacterial and Fungal Populations Colonizing the Scalp

**Figure 2:**
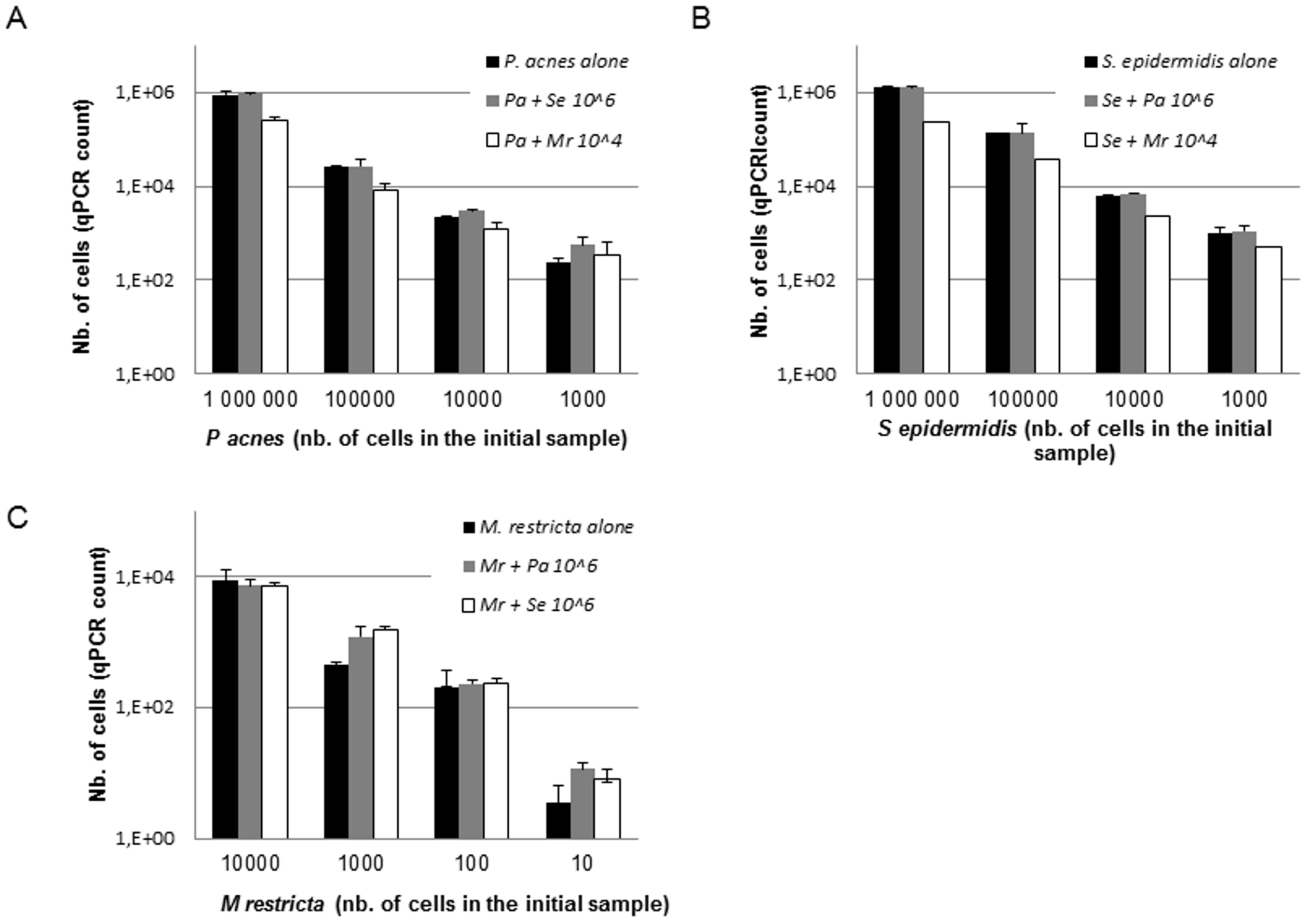
Q-PCR quantification of artificial mixtures containing genomic DNA extracted from *S. epidermidis* and *P. acnes* or *M. restricta* in various ratios. (A) quantification of *P. acnes* alone or in combination with *S. epidermidis* (ratios 1∶1; 1∶10; 1∶100 and 1∶1000) or *M. restricta* (ratios 1∶0.01; 1∶0.1; 1∶1 and 1∶10); (B) quantification of *S. epidermidis* alone or in combination with *P. acnes* or *M. restricta* in the same ratios and (C) quantification of *M. restricta* alone or in combination with *P. acnes* or *S. epidermidis* (ratios 1∶100; 1∶1000; 1∶10000 and 1∶100000). Q-PCR quantification showed the same cell count when the three microorganisms were analyzed alone or in a mixture with the other major skin contaminants.

Since three major microbial species were found on the scalps, their quantitative distribution was analyzed among individuals with or without dandruff. The analysis was performed on two distinct population samples at two times of the year for a better statistical validation of the results. These species were quantified by Quantitative-PCR (Q-PCR) using specific primers and TaqMan MGB probes targeting a specific region of the bacterial 16S rDNA sequences and the fungal ITS-28S rDNA sequences. The PCR primers used for quantification (Table S3) were specific of the *Propionibacterium* and *Staphylococcus* genera but since *P. acnes* and *S. epidermidis* accounted for >99% of all *Propionibacterium* and *Staphylococcus* species identified, we considered in our analysis that the counts of *Propionibacterium* and *Staphylococcus* corresponded to *P. acnes* and *S. epidermidis.* The 28S primers used for *Malassezia* were specific of the genus whereas the ITS primers permitted species discrimination, especially the accurate identification of the species *M. restricta and M. globosa*. Similarly, it was verified that the *M. restricta* primers did not amplify DNA from other *Malassezia* species including *M. globosa* and that there is no bias in DNA extraction or cloning of genomic DNA between *M. restricta* and *M. globosa* (data not shown). *M. globosa* was either not detected or not significant when checked by qPCR in 38 samples (data not shown); so, this species was not included further in the statistical analysis. No cross-reactivity was seen for *P. acnes* and *S. epidermidis* with other possible cutaneous microorganisms since none of the species of *Streptococcus*, *Corynebacterium*, *Acinetobacter* and *Malassezia* tested were amplified in our PCR assay (data not shown). In addition, using artificial mixtures of *S. epidermidis* and *P. acnes* or *M. restricta* in various ratios ranging from 1∶1 to 1∶1000, the same cell count was obtained when the three microorganisms were analyzed alone or in combination (Figure 2), indicating that our Q-PCR quantification method was reliable for the quantification of mixtures of the major microbial species found in the scalp.

**Figure 3:**
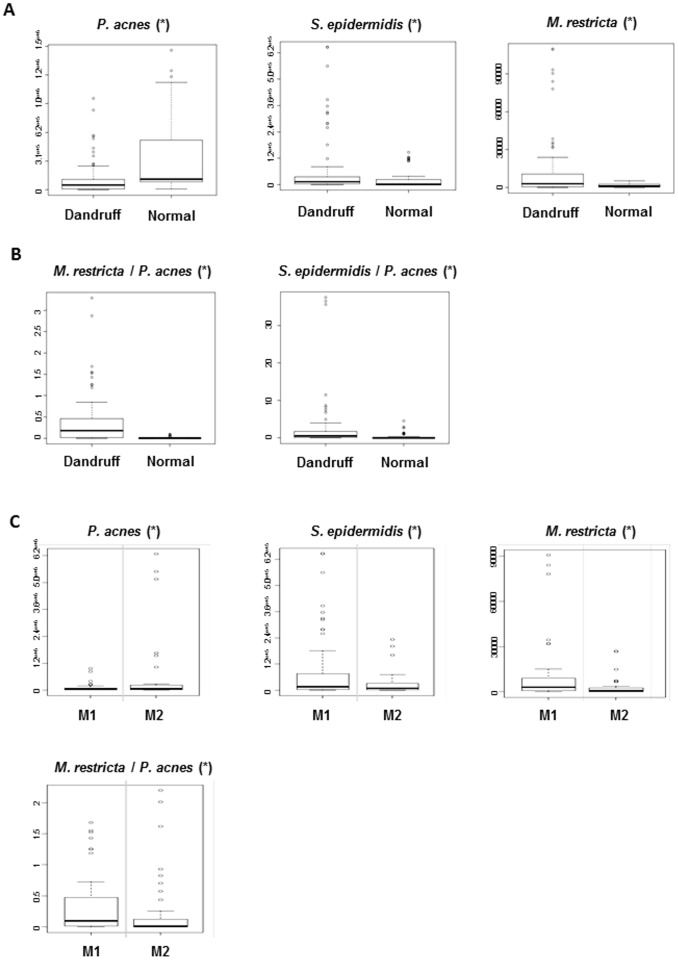
Quantification of the three major microbial species found on the scalp surface by Q-PCR. Box plots comparing the density of *P. acnes*, *S. epidermidis* and *M. restricta* found on the scalp surface (in number of cells per cm^2^ of the scalp surface detected by Q-PCR) - (A) Variations of the microbial populations between nineteen subjects from Set-1 and thirty subjects from Set-2 accounting for a total of 29 individuals with dandruff and 20 controls without dandruff; (B) Ratios *M. restricta/P. acnes and S. epidermidis/P. acnes* were significantly higher in dandruff scalps compared to non-dandruff scalps and (C) Intra-individual variations of the microbial populations within the 20 dandruff volunteers of Set-2. Box plots comparing the density of *P. acnes*, *S. epidermidis* and *M. restricta* quantified by Q-PCR on areas M1 (dandruff area) and M2 (non-dandruff area). *M. restricta*, *S. epidermidis* and *M. restricta/P. acnes* ratio were significantly higher and *P. acnes* incidence was lower in dandruff areas (M1) compared to non-dandruff areas (M2). Asterisk indicates a significant statistical difference (p<0.01). Note that the PCR primers used for quantification of the *Propionibacterium* and *Staphylococcus* were only genus-specific but since *P. acnes* and *S. epidermidis* accounted for 99% of all *Propionibacterium* and *Staphylococcus* species identified respectively, it was considered that the counts of *Propionibacterium* and *Staphylococcus* populations corresponded mainly to *P. acnes* and *S. epidermidis.*

Distance-based ANOVA analysis of the differences in the number of bacterial and fungal cells showed a significantly lower amount of *P. acnes* (from 3.5 10^5^ to 1.4 10^5^ cells/cm^2^, *p* = 0.002), a significantly higher amount of *M. restricta* (from 1.6 10^3^ to 1.3 10^4^ cells/cm^2^, *p* = 0.04) and *S. epidermidis* (from 2.1 10^4^ to 6.7 10^4^ cells/cm^2^, *p* = 0.02) in dandruff scalps than in non-dandruff scalps (Figure 3A). Dandruff was also associated with changes in the proportion between fungal and bacterial populations. The ratio *M. restricta/P. acnes* was significantly higher (*p* = 0.005) in subjects with dandruff (mean ratio = 0.37) as compared to individuals without dandruff (mean ratio = 0.012) (Figure 3B). In the bacterial communities, the *S. epidermidis/P. acnes* ratio was also significantly higher in the subjects with dandruff as compared to non-dandruff subjects (mean ratios were equal to 2.56 and 0.33, respectively; *p = *0.004) (Figure 3B).

These differences were confirmed intra-individually, within a panel of dandruff subjects, when the microbial population of an area (M1) with dandruff was compared to that of an area (M2) without dandruff from the same scalp (Figure 3C). The differences in the microbial populations between the samples collected in M1 and M2 on the same individual were similar to the differences observed among the subjects with or without dandruff: M1 areas exhibited a significantly higher incidence of *M. restricta* (from 1.9 10^3^ in M2 vs 7.0 10^3^ cells/cm^2^ in M1, *p* = 0.0006) and *S. epidermidis* (from 3.1 10^4^ in M2 vs 6.7 10^4^ cells/cm^2^ in M1, *p* = 0.002) and a lower incidence of *P. acnes* (from 3.4 10^5^ in M2 to 1.4 10^5^ cells/cm^2^ in M1, *p* = 0.002). Accordingly, a higher *M. restricta/P. acnes* ratio (mean values 0.18 and 0.33, respectively; *p* = 0.03) was observed in the samples collected in M2 compared to M1.

## Discussion

This study is the first comprehensive comparison of bacteria and fungi in dandruff subjects using culture-independent methodologies. The data presented here showed that the major fungal species found in the scalp of the French population is *M. restricta* and that dandruff is associated to an increase in the amount of this species. With the exception of an older and unrefined non-exhaustive study by McGinley *et al.* [15] in 1975 wherein molds were not quantified, the bacterial colonization of the dandruff scalp has not been investigated since the generally accepted rule was that dandruff was exclusively associated to the presence of *M. restricta* and *M. globosa* [9], [16], [17]. Identification of the cutaneous bacterial species has been often linked to skin diseases but the role of a mixed bacterial population on clinical skin syndromes has been poorly investigated [18]. In our study, *P. acnes* and *S. epidermidis* were the two major bacterial species found in dandruff or non-dandruff scalps. The presence of these two major bacterial species has been previously reported on human skin microbiota, with *P. acnes* being shown to be predominant in sebaceous rich body sites [19]. The present data show that in contrast to other studies dandruff is not only associated to the higher incidence of one particular *Malassezia* species but also to differences in the balance between the fungal and bacterial populations on the scalp [15], [16]. Such a finding and the ability to quantify quickly and accurately by a Q-PCR method the three species associated to the occurrence of dandruff should be useful for the development of new anti-dandruff strategies. A modification of the scalp microbial ecology either due to chemicals or changes in the host skin general physiology may improve the dandruff situation of the scalp. For dandruff treatment this is a new concept and the read out of this new managing strategy will be based on the quantification of the three targeted species.

Identification of the fungal species truly associated with dandruff or even inhabiting the normal scalp surface has been controversial in the past studies by Mc Ginley *et al*. (1975), Gemmer *et al*. (2002), Lee *et al*. (2006), Ashbee, *et al.* (2007) and Park *et al.* (2012) [9], [11], [15], [20], [21]. Several issues may be responsible for that: (i) problems in the isolation of the fungi and/or difficulties to grow them; (ii) uncertainty in the dermatological diagnosis and uncompleted or erroneous species identification especially since the nomenclature of *Malassezia spp*. has continuously evolved over the last 20 years; (iii) different origin of the sampled populations (North America, Korea or France) and it is known that the environment or ethnicities and food may influence the composition of a microbiome in the oral cavity, vagina and gut [22]–[24] and (iv) the identification techniques used in the different studies were different. The culture-independent methods are, to date, the most accurate to identify microbial scalp inhabitants since direct characterization of DNA from scalp samples avoid any bias as in the culture method which has been shown to be often a real problem for the identification of scalp microbial inhabitants. Discrepancies observed between our study and the mycobiota analysis reported by Park *et al.* (2012) could be explained by a different origin of the subjects or a different selection of primers sets for amplification of the ribosomal DNA [11]. Moreover, the three major fungal genera identified in their study (*Didymella, Acremonium and Filobasidium*) are the common airborne saprotrophic species which raised some concerns on the conclusion of the study. In addition, in contrast to the present report, Park’s recent study only focused on fungi and did not consider bacteria.

Although it has not been investigated in our study, it appears that the diversity of the species identified in the scalp was higher in dandruff scalps than in non dandruff scalps. Although this can be just a consequence of higher bacterial and fungal counts in the dandruff scalps, it can also indicate a higher propensity for the scalp of dandruff subjects to be colonized by a wide variety of scalp microbial contaminants. It will be now essential to study the specific interactions within this microbial community and the ability of the microbiota to interact with the two other etiologic facets of dandruff, which are host susceptibility and sebum metabolization [17]. A metagenomic study of a larger panel of volunteers would certainly help our understanding of the interactions of these microbial communities during dandruff formation.

## Materials and Methods

### Subject Recruitment

The study was conducted in compliance with the World Medical Association Declaration of Helsinki, national and EU regulations and L’Oréal Research and Innovation’s procedures based on ICH guidelines for Good Clinical Practice. According to the national “Arrêté du 11 mai 2009 relatif aux définitions de certaines catégories de recherches biomédicales”, this non-interventional study without tested product nor invasive assessment method did not require Regulatory Approval. However, this study has been approved by L’Oréal’s Ethic’s Group (studies number: 1) 2010-0152 and 2) ACR/PASFLO/1111). All volunteers received verbal and written information concerning the study in accordance with the applicable local regulations, guidelines and the current SOP. This information explained the nature, purpose and risks of the study and emphasized that participation in the study was voluntary and that the volunteer might withdraw from the study at any time and for any reason. The volunteer’s written informed consent to participate in the study was obtained prior to any study related procedure being carried out. All data was analyzed anonymously and steps were taken to protect the identities of all participants. According to national law “Informatique et liberté” dated January 6th 1978, modified by law No 94–548 dated July 1st 1994, and law n° 2004–801 dated August 6th 2004, the subject database is declared to the “Commission Nationale de l’Informatique et des Libertés” (the national commission of data processing and freedom)”.

### Sample Collection

Three weeks before sampling, the volunteers who participated in this study were given the same particular neutral shampoo (without any treating agent of perfume, similar to “DOP Ultra Doux”) to be used three times a week at home to avoid any bias in the results due to shampoo. Volunteers were not allowed using any other product on their hair or scalp during this 3-week wash-out period. The last shampoo was performed three days before the sampling procedure. The presence or absence of dandruff in the scalp of the subjects was assessed following Van Abbe’s method with modifications [25]: dandruff score equal to 0, 1, 2 or 3 was assigned as compared to reference pictures (Figure S1) on eight different sections of the scalp examined. The final score was the average of these eight values. Inclusion criteria were: a) for Dandruff volunteers, clinical aspect was determined as score 1, 2 or 3, and b) for control volunteers, no dandruff was observed and score was equal to zero as described in Table S4 and Figure S1.

For sample collection, we used the swab method as reported previously [19], [26] with few modifications to sample the scalp surface. A 16 cm^2^ area was sampled as follows: 8 segments of 4 cm length were successively isolated by separating hair fibers with a comb. Sterile cotton swabs were soaked in the tube containing 5.0 ml of NaCl (0.15 M)–Tween 20 (0.1%) solution and rubbed along one segment by making four passages. The same procedure was repeated on each of the 8 lines to cover the total surface in a non-overlapping manner. The head of each swab was cut from the handle, placed into the tube containing the NaCl-Tween solution. Scalp samples were stored at 4°C and processed for DNA isolation within 24 h. As negative controls, sterile cotton swabs were cut from the handle, placed into NaCl–Tween 20 solution (5.0 ml) and further processed according to identical conditions without any contact with scalp.

Forty-nine volunteers (22–63 years old; 40% male) were recruited to provide samples. Two independent sets of samples were collected in May 2010 for Set-1 and March 2011 for Set-2. Set-1 consisted of samples from 10 non-dandruff and 9 dandruff subjects which were analyzed for identification of the microbial population followed by Q-PCR quantification. Set-2 gathered samples from 10 non-dandruff and 20 dandruff-affected subjects. In addition, each dandruff scalp from Set-2 was collected in two areas: an area with dandruff (M1) and an area without dandruff (M2). Characteristics of the selected dandruff and control subjects are shown in Table S1 and S2.

### Fungal and Bacterial DNA Extraction

The protocol for DNA isolation was as follows: 2 ml of bacterial-fungal cells suspension from the scalp were pelleted by centrifugation for 20 min at 13500 rpm. The collected cells were resuspended in 400 µl of lysis buffer (20 mM Tris-HCl, 250 mM NaCl, 25 mM EDTA, 1%SDS (w/v), 1%Triton×100 (w/v); pH 8.0) containing 5 µl of proteinase K (10 mg/ml, Roche) and incubated for 16 h at 55°C, then for 5 min in a boiling water-bath. Cells were transferred to screw-capped tubes, 250 µl of glass beads (0.17–0.18 mm, Sartorius) were added and the samples were broken in a FastPrep (MP Biomedicals) by making three cycles of vortex for 60 s, speed 6.0. Supernatants were further supplemented with phenol and chloroform (400 µl each) and vortexed. After centrifugation for 20 min at 13500 rpm, the aqueous phase was removed and treated with an RNAase (2 µl, 10 mg/ml, Roche) for 3 h at 37°C. DNA was finally precipitated by adding ammonium acetate (5 mM, final concentration) and pure ethanol (1 ml) (at −20°C for 24 h). The resulting DNAs were pelleted by centrifugation (for 20 min at 13500 rpm), washed with ethanol 70% (700 µl) and finally suspended in 40 µl of ultra-pure DNAse-RNAse free water. DNA content was measured by Qubit dsDNA HS kit (Invitrogen). Our DNA extraction method was validated since genomic DNA extraction of a range of species tested separately gave similar amount of DNA per cell (Table S5). In particular, DNA of the same number of cells (10^6^ cells) of *M. restricta* strains (CBS7877) and of *M. globosa* strains (CBS7874) were extracted with this protocol. The same amount of DNA per cell was recovered for both *Malassezia* species (Table S5). In addition, in the nineteen volunteers (N1–N10 and D1– D9), the DNA quantification by using Q-PCR or by cloning and sequencing the ribosomal unit regions (16S and 28S-ITS) gave similar values.

### PCR Amplification

For each scalp sample from Set-1, two different PCR products were prepared from genomic DNA. The fungal ITS1-5.8S-ITS2 and part of the 28S (D1/D2) region were amplified with universal primers ITS1f and TW13 (Table S3, [27]). 16S rDNA was amplified using the sets of primers: CIP-pA and CIP-pH (Table S3, [19]). Both PCR products (∼1,500 bp) were then cloned individually. Amplification method was as follows: 5.0 µl of 10× buffer (Amersham Bioscience), 5.0 µl of dNTP mix (25 mM each, Roche), 1 µl of each primer (10 µM), 1 µl of DMSO, 1 µl of genomic DNA (20 ng) and 0.3 µl of rTaq Polymerase (Amersham) qsp 50 µl of water. For thermocycling, initial denaturation at 95°C for 5 min, followed by 35 cycles of a 60-sec 95°C denaturation, 60-sec annealing at 60°C, and 1.5 min elongation at 72°C, all followed by final extension for 15 min at 72°C. PCR products were purified using Qiaquick purification kit (Qiagen) as per the manufacturer’s instructions. As a control the same amount of genomic DNA of each strains *M. restricta* and *M. globosa* were mixed and PCR amplified using universal primers ITS1f and TW13 (Table S3, [27]) PCR fragments was cloned further as described below.

### Cloning and Sequencing

Fresh purified PCR products were cloned into the pCR2.1-TOPO vector (Invitrogen) as per manufacturer instructions using 1 µl of PCR product (20 ng DNA). Plasmid DNA purifications were performed using the Montage Plasmid Miniprep96 kit (Millipore). Sequencing reactions were performed with M13 Forward and Reverse primers using ABI Prism BigDye Terminator cycle sequencing-ready reaction kits and run on an ABI 3730×l Genetic Analyzer (Applied Biosystems). Base calling and quality clipping of the sequence traces were done using the script Assembler Tool Kit [28]. After excluding low quality sequences, 2,122 bacterial and 2,225 fungal sequences (∼1500 bp) of 16S rDNA and ITS-28S rDNA, respectively (which corresponded to 200 sequences in average per subject), were analyzed further at the phylum, genus, and species levels. For bacterial identification, the Ribosomal Database Project – II release 10, update 29 [29]) was used. For a first line of identification of fungal species, the Silva database was used [30]. To refine fungal identification since no 28S or ITS database existed for fungal species found on human skin, a custom database was designed with a selection of ITS and 28S fungal sequences of skin fungi [9], [26], [31]–[33]. Distribution of the 4,347 sequences obtained from the genomic DNA of the 19 subjects from Set-1 and 30 subjects from Set-2 are presented in Table S4. Accession numbers of highest homology matches used for the confirmation of identity of major bacterial and fungal species found in this study are presented in Table S6. Moreover, we demonstrated that there was no experimental bias when cloning genomic DNAs of *M. globosa* or *M. restricta;* we could recover equal amounts of *M. globosa* and *M. restricta* after PCR cloning of a DNA mix (in 1∶1 ratio) from both the species. Confirmation experiments were performed by PCR using specific primer pairs Mrest-F and Mrest-R or Mglo-F and Mglo-R (Table S3) to detect *M. restricta* species and of *M. globosa* species, respectively.

### Stability of Bacterial and Fungal Cell Suspension and Scalp Samples

Stability of DNA under storage at 4°C or −20°C for 24 h was investigated using scalp samples and also *M. restricta*, *P. acnes* and *S. epidermidis* cells grown *in vitro* and resuspended in the sampling buffer (10^7^cells/ml). Each sample was separated into three equal fractions. First fraction was extracted directly as described above, second fraction was extracted after 24 h at 4°C and third fraction was extracted after 24 h at −20°C. To assess the amount of bacterial and fungal genomic DNA extracted after the storage step, PCR amplification was performed by using universal primers ITS1f and TW13 or primers CIP-pA and CIP-pH (Table S3), followed by quantification of the PCR products by using Qubit dsDNA HS kit (Invitrogen). We obtained 25% reduction of the fungal PCR product after storage at −20°C, while there was no significant decrease in the amount of fungal and bacterial products after 24 h storage at 4°C. For the three strains, the amount of genomic DNA extracted was measured directly by using Qubit dsDNA HS kit (Invitrogen). Results showed equivalent amount of DNA recovered without storage and after storage for 24 h at 4°C. All the results were obtained in triplicate. Further, all the volunteer’s samples were stored at 4°C and their genomic DNA was extracted within 24 h. Moreover, the effect of cotton swabs on PCR reaction has been tested. DNA extraction of the three major species *M. restricta*, *P. acnes* and *S. epidermidis* has been performed in presence or absence of cotton swab and DNA content was measured by Qubit dsDNA HS kit (Invitrogen) after amplification using specific primers (Table S3). Results showed equivalent amount of DNA recovered with or without cotton swab.

### Quantitative PCR

The three major species identified in the scalp environment, *P. acnes*, *S. epidermidis* and *M. restricta*, and also the *M. globosa* species were quantified with Quantitative-PCR using specific primers and TaqMan MGB probes targeting a specific region of the bacterial 16S rDNA sequences or the fungal ITS-28S rDNA sequences. For the quantification of *S. epidermidis*, Staph-P probe [34] was used in combination with a set of new primers (Table S3). For *P. acnes,* new primers and probes were designed. All new primers and probes were designed by using primer express 4 (Applied Biosystem) and the corresponding bacterial 16S rRNA sequences obtained in this study. The 28S primers used for *Malassezia* were specific of the genus whereas the ITS primers permitted species discrimination and especially the accurate identification of the species *M. restricta* and *M. globosa* [35] (Table S3). The reaction mix consisted of 20 µl of TaqMan Universal Master Mix II without UNG (Applied Biosystem), 200 nM of each primer (Table S3), 250 nM TaqMan probe (Applied Biosystem) and DNA (0.5–5 ng). Amplification and detection were performed with the iCycler iQ (BIO-RAD) with the following cycle parameters: 55°C for 2 min, 95°C for 10 min, and 40 cycles of 95°C for 30 sec and 55°C for 30 sec, for *Malassezia* sp. or 55°C for 2 min, 95°C for 10 min, and 40 cycles of 95°C for 30 sec and 55°C for 45 sec for bacterial species. Every sample was run in triplicate. It was verified that there was a direct linear correlation between the concentration of *M. restricta*, *M. globosa*, *P. acnes* and *S. epidermidis* cells (between 10^2^ to 10^7^ cells) and the cycle threshold (Ct) values. The regression lines were respectively, Ct = −3.2× log(number of cells)+39 (r^2^ = 0.995) for *M. restricta*, Ct = −3.6× log(number of cells)+9 (r^2^ = 0.995) for *P. acnes* and Ct = −3.0×log(number of cells)+38 (r^2^ = 0.97) for *S. epidermidis*.

### Statistical Methods

ANOVA was used for statistical analysis in this study. The effect of dandruff status on the number of bacterial and fungal cells was tested with Type III Sum of Squares. Normality of the residuals and homogeneity of the variance were visually checked on plots. All statistical analysis were calculated by using R software [36].

## Supporting Information

Figure S1 **Dandruff scoring according to the modified Van Abbe’s method. A) Score 0, no dandruff.** B) Scalp with dandruff: 1 minimal level of dandruff observed and 3, highest level of dandruff observed.
(TIF)

Table S1 **Distribution of the bacterial 16S rDNA sequences from 19 subjects (N1-10, controls without dandruff; D1-9 subjects with dandruff).**
(DOCX)

Table S2 **Distribution of the fungal ITS-28S rDNA sequences from 19 subjects (N1-10, controls without dandruff; D1-9 subjects with dandruff).**
(DOCX)

Table S3 **Sets of primers used in this study and primers and probes that were used to quantify** ***M. restricta*** **,** ***M. globosa*** **,** ***Propionibacterium sp.*** **and** ***Staphylococcus sp*** **.**
(DOCX)

Table S4 **Distribution of the 4,347 sequences obtained by cloning and sequencing PCR products from the genomic DNA of the 19 subjects from Set-1 and characteristics of the 30 subjects from Set-2.**
(DOCX)

Table S5 **Genomic DNA isolation of individual bacterial and fungal species: list of the strains used to establish the DNA extraction method and genomic DNA amount obtained for each species.**
(DOCX)

Table S6 **GenBank accession numbers of major bacterial and fungal species found in this study.** Accession numbers of highest homology matches used for the confirmation of identity are presented.
(DOCX)
