# Correction: Dandruff Is Associated with Disequilibrium in the Proportion of the Major Bacterial and Fungal Populations Colonizing the Scalp

**DOI:** 10.1371/annotation/bcff4a59-10b7-442a-8181-12fa69209e57

**Published:** 2013-10-16

**Authors:** Cécile Clavaud, Roland Jourdain, Avner Bar-Hen, Magali Tichit, Christiane Bouchier, Florence Pouradier, Charles El Rawadi, Jacques Guillot, Florence Ménard-Szczebara, Lionel Breton, Jean-Paul Latgé, Isabelle Mouyna

There is an error in the last sentence of the section “The Presence of Dandruff is Associated with Disequilibrium in the Proportion of the Major Bacterial and Fungal Populations Colonizing the Scalp,” of Results.

The correct sentence should read “Accordingly, a higher M. restricta /P. acnes ratio (mean values 0.33 and 0.18, respectively; p=0.03) was observed in the samples collected in M1 compared to M2.”

