# Supplementary figures and images for: Dandruff Is Associated with Disequilibrium in the Proportion of the Major Bacterial and Fungal Populations Colonizing the Scalp
Source: PLoS One. 2013 Mar 6;8(3):e58203. doi: 10.1371/journal.pone.0058203 (PMC3590157; doi:10.1371/journal.pone.0058203)

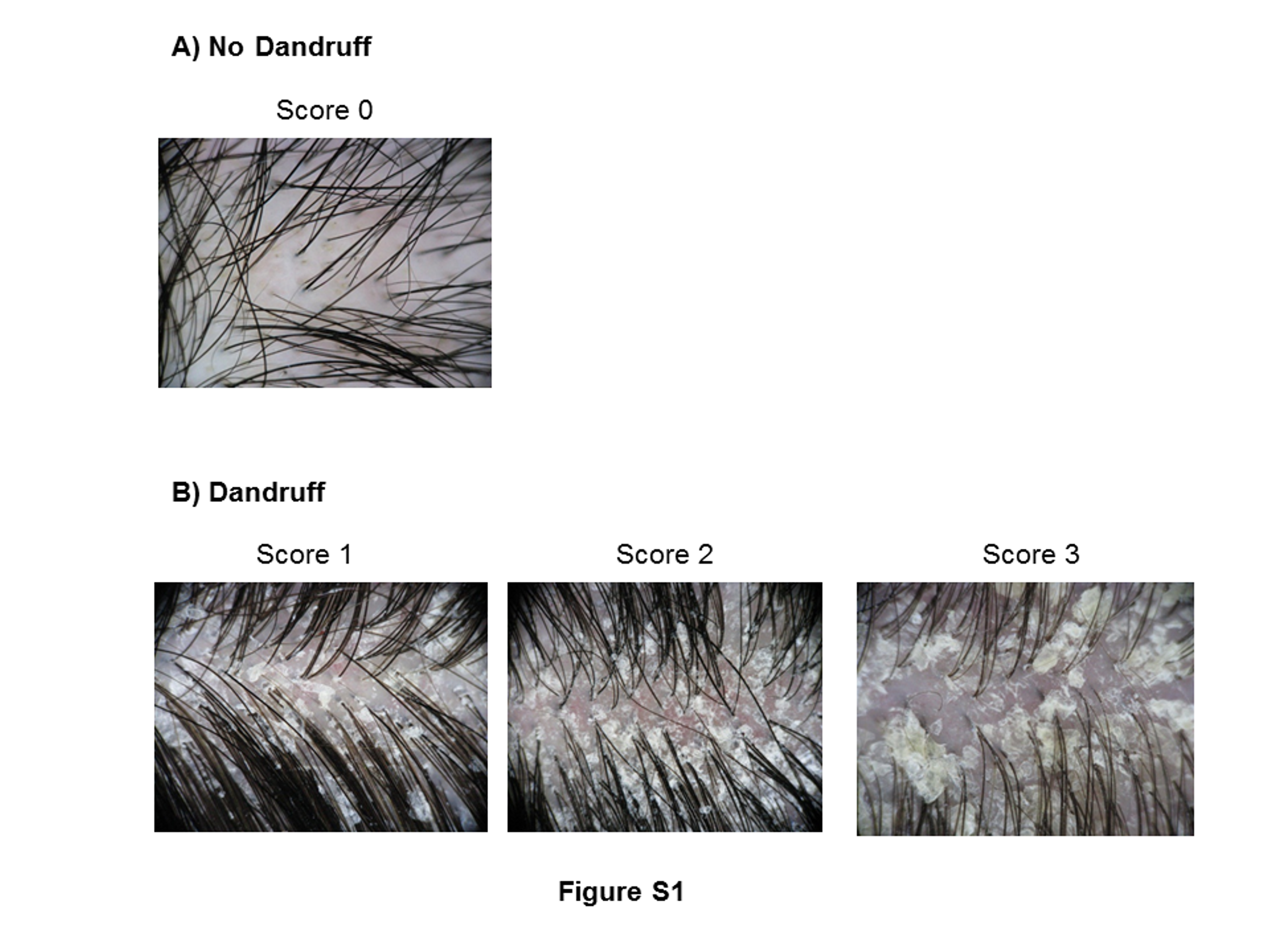

Supplement: Figure S1 — Dandruff scoring according to the modified Van Abbe’s method. A) Score 0, no dandruff. B) Scalp with dandruff: 1 minimal level of dandruff observed and 3, highest level of dandruff observed. (TIF) [file pone.0058203.s001.tif]
