# Supplementary material for: Dandruff Is Associated with Disequilibrium in the Proportion of the Major Bacterial and Fungal Populations Colonizing the Scalp
Source: PLoS One. 2013 Mar 6;8(3):e58203. doi: 10.1371/journal.pone.0058203 (PMC3590157; doi:10.1371/journal.pone.0058203)
Supplement: Table S2 — Distribution of the fungal ITS-28S rDNA sequences from 19 subjects (N1-10, controls without dandruff; D1-9 subjects with dandruff). (DOCX) [file pone.0058203.s003.docx]

**Table S2**: Distribution of the fungal ITS-28S rDNA sequences from 19 subjects (N1-10, controls without dandruff; D1-9 subjects with dandruff).
