# Supplementary material for: Dandruff Is Associated with Disequilibrium in the Proportion of the Major Bacterial and Fungal Populations Colonizing the Scalp
Source: PLoS One. 2013 Mar 6;8(3):e58203. doi: 10.1371/journal.pone.0058203 (PMC3590157; doi:10.1371/journal.pone.0058203)
Supplement: Table S3 — Sets of primers used in this study and primers and probes that were used to quantify M. restricta , M. globosa , Propionibacterium sp. and Staphylococcus sp . (DOCX) [file pone.0058203.s004.docx]

**Table S3:** Sets of primers used in this study and primers and probes that were used to quantify *M. restricta*, *M. globosa*, *Propionibacterium sp.* and *Staphylococcus sp*.

| **Primers and probes** | **Primer sequence (5’-3’)** | **Reference** |
| --- | --- | --- |
| **Fungal ITS-28S rDNA** | | |
| TW13 (Forward primer) | GGTCCGTGTTTCAAGACG | [25] |
| ITS1-f (Reverse primer) | CTTGGTCATTTAGAGGAAGTAA | [25] |
| **Bacterial 16S rDNA** | | |
| CIP-pA (Forward primer) | AGAGTTTGATCATGGCTCAG | [26] |
| CIP-pH (Reverse primer) | AAGGAGGTGATCCAACCGCA | This study |
| **All *Malassezia* species** | | |
| Mala-F (Forward primer) | CTAAATATCGGGGAGAGACCGA | [32] |
| Mala-R (Reverse primer) | GTACTTTTAACTCTCTTTCCAAAGTGCTT | [32] |
| Mala-MGB (probe) | FAM-TTCATCTTTCCCTCACGGTAC-MGB | [32] |
| ***M. restricta*** | | |
| Mrest-F | GGCGGCCAAGCAGTGTTT | [32] |
| Mrest-R | AACCAAACATTCCTCCTTTAGGTGA | [32] |
| Mrest-MGB | FAM-TTCTCCTGGCATGGCAT-MGB | [32] |
| ***M. globosa*** | | |
| Mglob-F | GGCCAAGCGCGCTCT | [32] |
| Mglob-R | CCACAACCAAATGCTCTCCTACAG | [32] |
| Mglob-MGB | FAM-ATCATCAGGCATAGCATG-MGB | [32] |
| ***Staphylococcus* genus** | | |
| 802F (Forward primer) | GGAGGAACACCRGTGGCGAA | This study |
| 862R (Reverse primer) | GCGTGAACTACCAGGGTATCTAA | This study |
| Staph-P (probe) | FAM-CTGTAACTGACGCTGATGTG-MGB | [31] |
| ***Propionibacteria* genus** | | |
| 591F (Forward primer) | CGAGCGTTGTCCGGATTT | This study |
| 654R (Reverse primer) | CACTTCCGACGCGATCAA | This study |
| Prop615-P (probe) | FAM-CGTAAAGGGCTCGTAGGT-MGB | This study |
