# Supplementary material for: Dandruff Is Associated with Disequilibrium in the Proportion of the Major Bacterial and Fungal Populations Colonizing the Scalp
Source: PLoS One. 2013 Mar 6;8(3):e58203. doi: 10.1371/journal.pone.0058203 (PMC3590157; doi:10.1371/journal.pone.0058203)
Supplement: Table S4 — Distribution of the 4,347 sequences obtained by cloning and sequencing PCR products from the genomic DNA of the 19 subjects from Set-1 and characteristics of the 30 subjects from Set-2. (DOCX) [file pone.0058203.s005.docx]

**Table S4:** Distribution of the 4,347 sequences obtained by cloning and sequencing PCR products from the genomic DNA of the 19 subjects from Set-1 and characteristics of the 30 subjects from Set-2.

| **Status** | **Designation** | **Dandruff flaking score** | **Age** | **Gender** | **Number of sequences studied** | |
| --- | --- | --- | --- | --- | --- | --- |
|  |  | **(swab site)^a^** |  |  | **16S rDNA inserts (~1500 bp)** | **ITS-28S rDNA inserts (~1500 bp)** |
| Controls (No Dandruff) | N1 | 0 | 44 | M | 129 | 119 |
|  | N2 | 0 | 31 | F | 110 | 161 |
|  | N3 | 0 | 22 | M | 98 | 126 |
|  | N4 | 0 | 53 | F | 202 | 173 |
|  | N5 | 0 | 50 | F | 131 | 88 |
|  | N6 | 0 | 26 | F | 108 | 90 |
|  | N7 | 0 | 38 | M | 131 | 98 |
|  | N8 | 0 | 60 | F | 103 | 117 |
|  | N9 | 0 | 51 | F | 102 | 99 |
|  | N10 | 0 | 55 | F | 127 | 102 |
|  | N11 | 0 | 47 | M | nd* | nd* |
|  | N12 | 0 | 39 | M | nd* | nd* |
|  | N13 | 0 | 57 | F | nd* | nd* |
|  | N14 | 0 | 59 | F | nd* | nd* |
|  | N15 | 0 | 28 | F | nd* | nd* |
|  | N16 | 0 | 54 | F | nd* | nd* |
|  | N17 | 0 | 56 | F | nd* | nd* |
|  | N18 | 0 | 32 | F | nd* | nd* |
|  | N19 | 0 | 47 | F | nd* | nd* |
|  | N20 | 0 | 47 | M | nd* | nd* |
| Dandruff | D1 | 1 | 35 | F | 139 | 133 |
|  | D2 | 2 | 42 | M | 79 | 133 |
|  | D3 | 2 | 44 | F | 99 | 118 |
|  | D4 | 2 | 63 | M | 127 | 125 |
|  | D5 | 2 | 57 | M | 58 | 72 |
|  | D6 | 2 | 24 | F | 88 | 117 |
|  | D7 | 2 | 50 | F | 98 | 98 |
|  | D8 | 3 | 38 | F | 101 | 119 |
|  | D9 | 3 | 61 | M | 92 | 137 |
|  | D10 | 1 | 43 | F | nd* | nd* |
|  | D11 | 2 | 38 | F | nd* | nd* |
|  | D12 | 2 | 38 | F | nd* | nd* |
|  | D13 | 2 | 35 | M | nd* | nd* |
|  | D14 | 3 | 54 | M | nd* | nd* |
|  | D15 | 2 | 39 | F | nd* | nd* |
|  | D16 | 1 | 51 | F | nd* | nd* |
|  | D17 | 3 | 35 | M | nd* | nd* |
|  | D18 | 3 | 54 | M | nd* | nd* |
|  | D19 | 2 | 56 | M | nd* | nd* |
|  | D20 | 3 | 42 | F | nd* | nd* |
|  | D21 | 3 | 55 | F | nd* | nd* |
|  | D22 | 3 | 30 | F | nd* | nd* |
|  | D23 | 2 | 36 | M | nd* | nd* |
|  | D24 | 2 | 53 | M | nd* | nd* |
|  | D25 | 2 | 50 | M | nd* | nd* |
|  | D26 | 3 | 28 | F | nd* | nd* |
|  | D27 | 1 | 38 | F | nd* | nd* |
|  | D28 | 3 | 51 | F | nd* | nd* |
|  | D29 | 3 | 43 | F | nd* | nd* |

^a^: Dandruff flaking score was determined for each swab site according to Fig S1 and reported in this table. For subjects D10 to D29, dandruff scores from M1 areas are presented in this table.

*: these samples were not submitted to cloning and sequencing and were analysed with qPCR directly.
