# Supplementary material for: Dandruff Is Associated with Disequilibrium in the Proportion of the Major Bacterial and Fungal Populations Colonizing the Scalp
Source: PLoS One. 2013 Mar 6;8(3):e58203. doi: 10.1371/journal.pone.0058203 (PMC3590157; doi:10.1371/journal.pone.0058203)
Supplement: Table S5 — Genomic DNA isolation of individual bacterial and fungal species: list of the strains used to establish the DNA extraction method and genomic DNA amount obtained for each species. (DOCX) [file pone.0058203.s006.docx]

**Table S5**: Genomic DNA isolation of individual bacterial and fungal species: list of the strains used to establish the DNA extraction method and genomic DNA amount obtained for each species.

|  | **Species** | **Strain*** | **Amount of cells** | **Genomic DNA (µg / µl)** |
| --- | --- | --- | --- | --- |
| Bacteria | *Acinetobacter junii* | CIP 64.5^a^ | 2 x 10^9 | 0.55 ± 0.08 |
|  | *Propionibacterium acnes* | CIP A179^a^ | 2 x 10^9 | 0.59 ± 0.10 |
|  | *Pseudomonas aeruginosa* | PAO3^a^ | 2 x 10^9 | 0.45 ± 0.06 |
|  | *Staphylococcus caprae* | CIP104000^a^ | 2 x 10^9 | 0.52 ± 0.05 |
|  | *S. epidermidis*  *Streptococcus mitis* | this study  CIP103335^a^ | 2 x 10^9  2 x 10^9 | 0.43 ± 0.06  0.66 ± 0.09 |
| Fungi | *Aspergillus fumigatus* | ATCC46645^c^ | 1 x 10^6 | 0.10 ± 0.01 |
|  | *Malassezia sympodialis* | this study | 1 x 10^6 | 0.12 ± 0.02 |
|  | *M. restricta* | CBS7877^b^ | 1 x 10^6 | 0.18 ± 0.04 |
|  | *M. globosa* | CBS7874^b^ | 1 x 10^6 | 0.16 ± 0.02 |

*Strains were cultivated as recommended by the provider library: ^a^ Biological Resource Center of Institut Pasteur (Institut Pasteur, Paris, France); ^b^ Centraalbureau voor Schmmelcultures (CBS, Utrecht, Netherlands). *A. fumigatus* was cultivated on 2% Malt agar slants for 7 days at 37°C.
