# Supplementary material for: Dandruff Is Associated with Disequilibrium in the Proportion of the Major Bacterial and Fungal Populations Colonizing the Scalp
Source: PLoS One. 2013 Mar 6;8(3):e58203. doi: 10.1371/journal.pone.0058203 (PMC3590157; doi:10.1371/journal.pone.0058203)
Supplement: Table S6 — GenBank accession numbers of major bacterial and fungal species found in this study. Accession numbers of highest homology matches used for the confirmation of identity are presented. (DOCX) [file pone.0058203.s007.docx]

**Table S6**: GenBank accession numbers of major bacterial and fungal species found in this study. Accession numbers of highest homology matches used for the confirmation of identity are presented.

| **Phylogenetic affiliation** | **Number of clones** | **Main accession number(s) retrieved** | **Database** |
| --- | --- | --- | --- |
| *Propionibacterium acnes* | 1044 clones | CP003084; CP002815; CP001977 | Genbank |
| *Staphylococcus epidermidis* | 838 clones | AF270147; CP000029 | Genbank |
| *Malassezia restricta* | 2023 clones | AY743636; AY743607; EU915456; AY387145; DQ789978; AY387241 | Genbank |
